# Urban income segregation and homicides: An analysis using Brazilian cities selected by the Salurbal project

**DOI:** 10.1016/j.ssmph.2021.100819

**Published:** 2021-05-17

**Authors:** Maria Izabel dos Santos, Gervásio Ferreira dos Santos, Anderson Freitas, J. Firmino de Sousa Filho, Caio Castro, Aureliano S. Souza Paiva, Amélia A. de Lima Friche, Sharrelle Barber, Waleska Teixeira Caiaffa, Maurício L. Barreto

**Affiliations:** a Center of Data and Knowledge Integration for Health (CIDACS), Brazil; b Faculty of Economics (PPGE) – Federal University of Bahia, Brazil; c Institute of Public Health (ISC) – Federal University of Bahia, Brazil; d Observatory for Urban Health in Belo Horizonte (OSUBH) – Federal University of Minas Gerais, Brazil; e Department of Epidemiology and Biostatistics – Drexel University Dornsife School of Public Health, Brazil

**Keywords:** Income segregation, Dissimilarity index, Homicides, Urban health

## Abstract

This paper investigates the associations of income segregation with homicide mortality across 152 cities in Brazil. Despite GDP increases, an important proportion of the Brazilian population experiences poverty and extreme poverty. Segregation refers to the way that different groups are located in space based on their socioeconomic status, with groups defined based on education, unemployment, race, age, or income levels. As a measure of segregation, the dissimilarity index showed that overall, it would be necessary to relocate 29.7% of urban low-income families to make the spatial distribution of income homogeneous. For the ten most segregated cities, relocation of more than 37% of families would be necessary. Using negative binomial models, we found a positive association between segregation and homicides for Brazilian cities: one standard deviation higher segregation index was associated with a 50% higher homicide rate when we analyze all the socioeconomic context. Income segregation is potentially an important determinant of homicides, and should be considered in setting public policies.

## Highlights

• The Dissimilarity index has a strong association with the number of homicides in large Brazilian cities.
• The more segregated cities are, the greater the indicators of inequality and poverty.
• The socioeconomic context matters for the reduction in the number of homicides.
• Brazil is one of the most violent countries in the world, therefore reducing inequality and segregation is urgent.

## 1 Introduction

One of the challenges faced by policy-makers worldwide has been to deal with the consequences of rapid and increasing urbanization. In different countries throughout Latin America and the Caribbean, about 80% of the population is living in urban areas; it is a dramatic increase from only 41.8%, in 1950 (UN, 2018). This transition brings important consequences regarding urban settlements, such as the increasing population living in slums, urban inequalities, segregation, and high levels of violence.

An important issue related to a fast and disorganized process of urbanization is income segregation. Segregation refers to the way that different population groups are located in space based on their socioeconomic status, whereby groups are defined by the education, unemployment, race, age, or income levels. Segregation, as a social phenomenon, started being investigated in the early 1920's, especially in the United States, by the so-called Chicago School including work by PARK and BURGESS (1925) who studied and attempted to explain the distribution of population across cities. Since then, a lot of research on this topic has been carried out, including addressing the particularities of developing regions and countries, such as Brazil (Barber et al., 2018; KING, 2009; Madalozzo, 2010; Marques, 2016; Rotondano, 2019).

As a multidimensional phenomenon, the effects of income segregation are differently felt by distinct social groups and have a collective and lasting impact on the lives of the most vulnerable groups through, essentially, the spatial segregation of poverty (Reardon and Bischoff, 2011). The authors also state that segregation implies that low-income families will generally live in neighborhoods with a lower average income than high-income families and this fact could be a result of perpetuation effect of an already existing socially discriminatory structure of inequality.

Income segregation and income inequality differ because inequality deals with how unequal resource allocation is in society among all individuals, with high inequality leading individuals to marginalization. On the other hand, segregation deals with spatial inequality in resource allocation. Socioeconomic segregation mainly affects the population of large cities, due to the lack of urban planning and the market effects on urban land and housing allocation (Bischoff and Reardon, 2013, pp. 1–43; Garcia-López and Moreno-Monroy, 2018; Reardon and Bischoff, 2011).

Although limited, available evidence suggests that segregation may weaken social connections and increase violence rates (Stafford et al., 2004). In a city with a high level of inequality, the spatial segregation may limit resources and services locally available specially to the poor, and decrease the gains from social interactions in the urban environment. Thus, low-income families are affected not only in the economic sense. Living in deprived neighborhoods within cities implies uneven results and disadvantages compared to high-income families. Stafford et al. (2004) conclude that socioeconomic segregation between neighborhoods is a factor that conditions important results regarding to employment and health-related risks, for instance.

Regarding Brazil, Marques (2016) examined the relationship between segregation, poverty and social connections in two large metropolises. The research concluded that sociability among individuals within cities is conditioned by variables such as housing, income and employment. Segregation can deep patterns of inequalities and income concentration already accentuated in Latin America and Brazil (Souza, 2018). Thus, it can be in a substantive pathway of the high number of homicides in Brazilian cities (Cerqueira et al., 2019, p. 116).

The high numbers and rates of homicides remain one of the majors health problems worldwide and especially in Latin America (Benjet et al., 2019; Yapp and Pickett, 2019). Brazil, in particular, had more than 63 thousand homicides in 2016 (Cerqueira et al., 2018), equivalent to four times the 16,913 deaths in the civil war in Syria in the same year (SNHR, 2016). According to Comelli, Angelovski and Chu (2018), the process of gentrification in the favelas of Rio de Janeiro, Brazil, increased socioeconomic conflicts. Moreover, police militarization has accentuated discrimination among residents unrelated to local organized crime. This finds suggests that violence affects vulnerable individuals in different ways since structural factors contribute to the growing of homicides in Brazilian cities.

Few studies have investigated the links between income segregation and violence or homicides (Votruba and Kling, 2009; Alonso and Hita, 2013; Logan and Parman, 2017; Cufré, 2019). Understanding how income segregation in cities relates to homicides is critical to inform public security policies and health. Therefore, we investigate the association between income segregation and homicides across the largest cities in Brazil. We hypothesized that higher segregation would be associated with higher homicide rates.

**Fig. 1:**
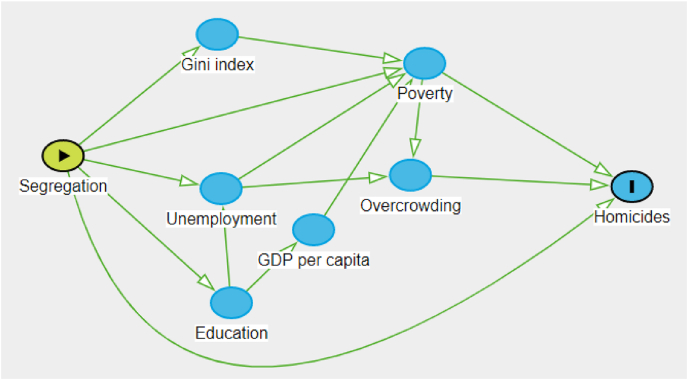
Directed Acyclic Graph of the associations between segregation and homicides.

Fig. 1 shows a Directed Acyclic Graph (DAG) used in this paper to represent the relationship between income segregation and homicides.^1^ Income segregation deepens inequalities, that contribute to social fragmentation, and worsen social problems such as the high level of homicides that characterizes many Latin American cities. There are multiple potential causal pathways between income segregation and homicides, and these are not mutually exclusive. We hypothesize that increasing segregation will increase the number of homicides, mediated by several socioeconomic and individual factors, namely income inequality, poverty, education, unemployment, overcrowding, and the GDP per capita.

We included a direct association between segregation and homicides to emphasize that there is a strong relationship between them (Comelli, Anguelovski and Chu, 2018; Cufré, 2019). However, there are several multi-step paths that can be taken between our exposure variable and the outcome. For instance, segregation accentuates inequality and poverty as opportunities between neighborhoods are unequal distributed (Ross, Nobrega and Dunn, 2001; Souza, 2001; Reardon and Bischoff, 2011). Another possible path is segregation directly impacting the educational level of residents which affects level of local or global income causing poverty and a major number of homicides (Williams and Collins, 2001; Watson, 2009; Logan and Parman, 2017). In these spaces, there is also greater unemployment which leads to worse housing conditions, such as overcrowding. All these factors can cause the increase of homicides (Stafford et al., 2004; Wilkinson, 2004; WHO, 2018).

These mediators are relevant, as analyzing the social context within cities is essential for elaborating political agendas. Moreover, society needs actions that will require collective efforts by institutions in order to deepen investigations and point out plausible solutions for reducing violence in urban centers.

In addition to this introduction, the paper is organized into 3 more sections. Section 2 presents the database, the method used to calculate the dissimilarity index, and the description of the negative binomial model. Results are presented in Section 3. Section 4 is aimed at the theoretical discussion. The paper concludes with a discussion of political implications related to segregation and homicides in large Brazilian urban centers.

## 2 Data and methods

The analytic dataset includes 152 Brazilian cities of 100,000 residents or more, as defined by the Urban Health in Latin America (SALURBAL, Spanish acronym) project. The project identified Latin American cities with more than 100,000 inhabitants across 11 countries. These cities are composed of one or more contiguous municipalities, as described in Quistberg et al. (2019). Data on socioeconomic, health, environment, and built environment characteristics have been assembled for each city. One of the aims of the SALURBAL project is to quantify how city and neighborhood factors such as infrastructure, segregation by income or education, pollution, transportation options, food availability, and violence can affect health and health inequalities in cities in Latin America. In this section, we describe the data (2.1), outline the method used to compute the segregation index – the dissimilarity index - (2.2), and the regression model used to analyze the relationship between segregation and homicides (2.3).

### 2.1 Data

The *outcome* variable is the total count of homicides in each city during the year 2010. The mortality dataset was originally obtained from the Brazilian Mortality Health Information System (SIM, Portuguese acronym) and it was harmonized and consolidated by the Data and Methods Core team of the SALURBAL project. Homicides were defined using Global Health Estimates (GHE) code 1580 (violence) and 1600 (Intentional injuries), which includes all deaths resulting from aggressions and its aftermath, specifically the international classification of diseases (ICD-10) categories X85 to Y099 and the Y871, and Y35. Mortality data were corrected for undercounting, using estimates of undercounting based on Campos de Lima and Queiroz (2014) method, as described previously for SALURBAL (Quistberg et al., 2019; Bilal et al., 2019).

The cross-sectional ecological analysis is based upon area-level linkage of homicide counts to 2010 population census data from the Brazilian Institute for Geography and Statistics (IBGE, Portuguese acronym). The Socioeconomic Status (SES) *variables* used in the regression analysis as potential city-level confounders and mediators are listed in Table 1.

**Table 1:** Potential SES variables at the city level to be used in the regression model, 2010

Variables	Definition	City level	Source
***Population***	Projected population	L1AD	SALURBAL
***Gini index***	Income inequality computed based on the household total income	L1AD	SALURBAL
***GDP per capita***	GDP/Population	L1AD	SALURBAL
***Complete secondary education***	Proportion of the population aged 25 or older who completed secondary education or above		SALURBAL
***Unemployment***	The unemployment rate among the total population 15 years or above in the labor force	L1AD	SALURBAL
***Poverty rate***	The proportion of the population living in households with household income below the national income poverty line	L1AD	SALURBAL
***Overcrowding***	Overcrowding: Proportion of households with more than 3 people per room	L1AD	SALURBAL

Source: Authors' elaboration.

### 2.2 Socioeconomic segregation measure (dissimilarity index)

The key explanatory variable is the income-based dissimilarity index henceforth referred to as the socioeconomic segregation index (SSI). We obtained the SSI by using income data of each census tract in a city. That is, there is an aggregation of spatial levels. The indicator is calculated taking into account residents in a specific census tract, then in the neighborhood and, finally, in the city.

The dissimilarity index by Duncan and Duncan (1955) is a measure of evenness, and it reflects the proportion of the population from each city that would have to be relocated to homogenize the urban area overall. The index ranges from 0 (complete integration) to 1 (complete segregation)^2^ (Iceland, Weinberg, and Steinmetz, 2002). Therefore, in this paper, the index is calculated according to the following formula:

(1) $$D=\;\frac{\sum_{i=1}^{n}\left[t_{i}\left|\left(p_{i}-P\right)\right|\right]}{\left[2TP\left(1-P\right)\right]}$$

In equation (1), n is the number of areas (census tracts) that make up each city. $t_{i}$ is the total households of area i. T is the total households, i.e. the sum of all, $t_{i}$. The number of poor households of area *i* is $x_{i}$ and X the sum of all $x_{i}$. So, $p_{i}$ is the ratio of $x_{i}$ to $t_{i}$ (proportion of area $i^{\text{′}}s$ poor households) and P is the ratio of X to T (proportion of the city area's households that is poor).

The dissimilarity index measures the evenness with which poor and non-poor households are distributed across the census tracts that make up a city. Following the process presented by Telles (1995) and Torres (2006) we used the 2 minimum wages cut-off criteria to define the low-income (poor) population. Thus, the poor population refers to households in which the head of the household has monthly income up to 2 official minimum wages.^3^

### 2.3 Statistical analysis

The outcome is the count of homicides in each city in the year 2010. Thus, we implement a set of binomial negative regression models to examine the relationship between segregation and homicides, considering a group of socioeconomic variables. All variables are aggregated at the city level, as defined by the SALURBAL project. The dataset is a cross-section for the year of 2010. The negative binomial regression is more adequate to obtain unbiased estimates since our outcome variable seems to be over-dispersed: the unconditional mean (247.2) of the number of homicides age-adjusted in 2010 is lower than its variance (351685.3).

Equation (4) presents the basic model, which corresponds to the standard negative binomial model, as described in Greene (2008):

(4) $$E\left[\mathrm{hom}icides_{i}|x_{i},\;\varepsilon_{i}\right]=\exp\left(\alpha +{x_{i}}^{\prime }\beta +\varepsilon_{i}\right)=\;h_{i}\gamma_{i}$$

where *i* represents each city, $x_{\text{i}}$ is the vector of covariates,^4^ $\varepsilon_{\text{i}}$ is the latent heterogeneity caused by the overdispersion, $\gamma_{\text{i}}$ is the conditional mean and ${\text{h}}_{\text{i}}=\text{exp}\left(\varepsilon_{\text{i}}\right)$ is assumed to have a one-parameter gamma distribution, G(θ,θ) with mean 1 and variance $\frac{1}{\theta }=\text{k}$.

All continuous independent variables, including the dissimilarity index, were converted in z-scores to simplify the interpretation, so that a change of 1 standard deviation (SD) in these predictors leads to an expected change of *β* units in the dependent variable, assuming all the other variables in the model are held constant.

## 3 Results

### 3.1 Descriptive and spatial analysis

Table 2 shows the descriptive statistics for our sample of cities divided by segregation quartiles. We observed some significant gradients across the quartiles. While the GDP per capita decreases, the Gini index, poverty, unemployment and homicide rates, consistently increase. In appendix B, we present a detailed correlation analysis of all variables used.

**Table 2:** Descriptive statistics by segregation quartiles.

Q1 (below 25%) *(N = 39)*	Mean (SD)	Variance	Q2 (50%) *(N = 37)*	Mean (SD)	Variance	Q3 (75%) *(N = 38)*	Mean (SD)	Variance	Q4 (100%) *(N = 38)*	Mean (SD)	Variance
Homicides rate	17.1 (11.9)	142.02		22.4 (18.4)	340.7		37.3 (22.6)	511.7		42.1 (23.0)	533.5
Population	228000 (137000)	1.87e+10		289000 (342000)	1.17e+11		517000 (647000)	4.19e+11		1860000 (3680000)	1.36e+13
GDP per capita	19412.8 (9890.8)	9.78e+07		16946.6 (8280.4)	6.86e+07		15388.9 (9571.9)	9.29e+07		14838.5 (11369.4)	1.29e+08
Gini	.51 (.02)	.001		.54 (.029)	.001		.55 (.031)	.001		.6 (.04)	.002
Poverty rate	15.2 (6.1)	37.44		20.5 (9.2)	84.6		30.0 (13.8)	191.7		36.0 (11.6)	136.3
Unemployment	6.3 (1.9)	3.7		7.9 (2.1)	4.6		9.8 (2.8)	7.8		11.3 (2.8)	8.0
Complete secondary	38.1 (4.7)	22.6		41.0 (5.2)	27.3		39.0 (7.1)	50.9		41.4 (7.1)	51.3
Overcrowd 3b	1.7 (1.6)	2.6		2.4 (1.4)	2.0		3.5 (2.8)	8.1		3.8 (2.5)	6.5

**Fig. 2:**
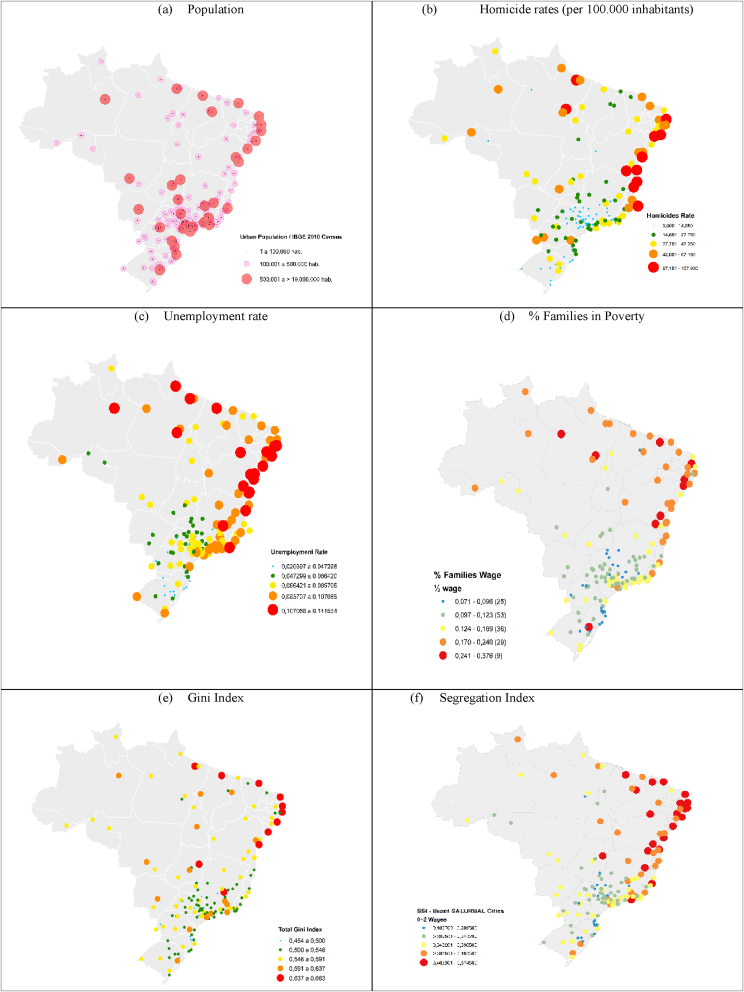
Selected indicators of Brazilian SALURBAL Cites, 2010.

The most populous cities in Brazil in the last Census in 2010 were São Paulo, Rio de Janeiro in the Southeast region, Salvador in the Northeast region, and Brasília in the Midwest region, with 19.8, 11.9, 3.1 and 3.2 million inhabitants, respectively. The main indicators for the regions can be consulted in Appendix A. Fig. 2a presents the population distribution of cities, over the country. The Southeast and Northeast regions include the majority of cities above 100 thousand inhabitants. The most populous cities also presented the largest number of homicides: Rio de Janeiro, 4.334; São Paulo, 3.769; and Salvador, 2.411. In terms of homicide rates adjusted by age (per 100.000 inhabitants), also the most violent cities were Rio de Janeiro (323), São Paulo (273), and Salvador (188) (Fig. 2b).

The cities with the highest homicides rates are mainly concentrated in the Northeast region, the country's most impoverished region. The Northeast region notably has the country's largest unemployment rates, as is shown in Fig. 2c. This region also presents the highest share of people living in the poverty situation (Fig. 2d).^5^ High unemployment can affect income distribution (Fig. 2e) and can deepen inequalities and segregation (Fig. 2f within cities.

### 3.2 Statistical analysis for the selected income socioeconomic segregation index

**Fig. 3:**
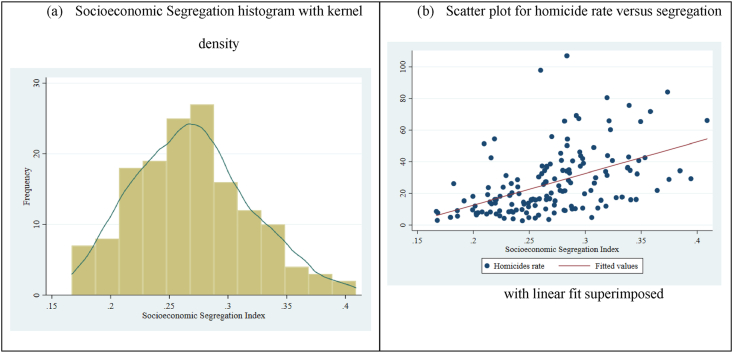
Socioeconomic segregation index based on total household wage with up to 2 minimum wages and a scatter plot for homicide rates versus segregation, in 2010.

Fig. 3 presents the distribution of the SSI for 152 Brazilian cities for the year 2010. The average index is 0.269 (from a minimum of 0.166 to a maximum of 0.408), which means achieving an even spatial distribution of low-income families (below twice the minimum monthly wage), in the large Brazilian cities, 26.9% of families would need to be relocated. SSI was positively correlated with the homicides rates (R^2^ = 0.25), as we show in Fig. 3b.

Table 3 presents the ranking for 20 cities with the highest SSIs. It is worth noting that 15 of them are located in the Northeast region, the country's poorest region.

**Table 3:** 20 most income segregated cities from the 151 Brazilian SALURBAL cities.

City	Region	State	SSI	Population	Gini	Homicide rates	Homicides (count)
1. João Pessoa	Ne	Pb	0.409	1049093	0.674	66.154	694.013
2. Aracaju	Ne	Se	0.394	856846	0.682	29.354	251.523
3. Brasília	Cw	Df	0.385	3235485	0.683	34.342	1111.135
4. Natal	Ne	Rn	0.375	1265118	0.645	28.907	365.703
5. Maceió	Ne	Al	0.374	1099695	0.649	84.120	925.064
6. Teresina	Ne	Pi	0.364	976798	0.636	21.882	213.739
7. Vitória de Santo Antão	Ne	Pe	0.358	323316	0.553	71.710	231.851
8. Recife	Ne	Pe	0.353	3588741	0.673	42.570	1527.714
9. Salvador	Ne	Ba	0.349	3371671	0.649	65.448	2206.689
10. Campina Grande	Ne	Pb	0.348	471572	0.589	40.780	192.306
11. Petrolina	Ne	Pe	0.346	508862	0.591	32.334	164.535
12. Sobral	Ne	Ce	0.345	190848	0.545	16.208	30.933
13. Teófilo Otoni	Se	Mg	0.341	138440	0.557	16.027	22.188
14. Rio de Janeiro	Se	Rj	0.340	11798818	0.629	34.611	4083.726
15. Ilhéus	Ne	Ba	0.339	194112	0.589	75.585	146.720
16. Fortaleza	Ne	Ce	0.339	3459235	0.650	43.059	1489.515
17. Garanhuns	Ne	Pe	0.338	132192	0.569	36.429	48.156
18. São Luis	Ne	Ma	0.338	1315122	0.652	36.037	473.937
19. Palmas	N	To	0.333	234217	0.596	17.691	41.435
20. Santos	Se	Sp	0.328	1664462	0.542	17.294	287.847

Note: Regions are abbreviated as: South (s); Southeast (se); North (n); Northeast (ne) and Midwest (cw).

**Fig. 4:**
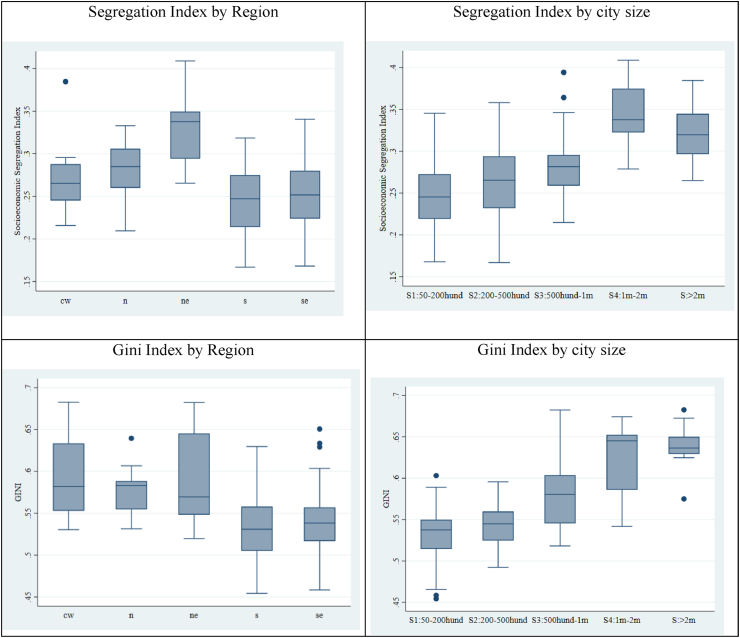
Socioeconomic Segregation and Income Inequality (Gini index) by macroregion and city size in Brazil (152 cities), 2010.

A descriptive analysis of SSI and Gini Index, for the 5 Brazilian macroregions and according to cities size, is shown in Fig. 4. As can be seen, income segregation and income inequality present similar trends by region and city size. The cities in the richest regions (South and Southeast) are the least segregated and also the least unequal, and in the poorest regions (North and Northeast) are the most segregated and also the most unequal. The Midwest region (intermediate income in the country) also is in an intermediate position. A pattern relating inequality and segregation also is verified when considering the size of the population in the cities. The larger the population in cities, the greater are the inequality and the spatial segregation.

When looking at the issues of regional inequality in Brazil, we observe that great disparities have perpetuated over time between the poorest and wealthiest regions of the country (Souza, 2018). Measuring the degree of inequality and socioeconomic segregation constitute current challenges for applied and social sciences, as they permeate areas of political and social conflict, violence, health-related issues, among many others (Marmot and Wilkinson, 2006, p. 376). Watson (2009), states that there is a close relationship between inequality and income segregation. The higher the inequality indicators, the greater the spatial segregation among residents since the poor are isolated in the most deprived neighborhoods in the city.

### 3.3 Regression results

Table 4 presents the estimation results according to equation (4), using the negative binomial model, controlling by the total city population. The results are presented in terms of incidence rate ratio (IRR). We estimate a model to capture the direct relationship between income segregation and homicides (Model 1) and a second one (Model 2) to include other socioeconomic variables' contributions in the causal relationship. We assume that there are several causal paths between the exposure and outcome (Fig. 1). These paths are affected by mediating, colliding or confounding variables that can reduce the direct impact of the relationship between segregation and homicides. Therefore, the first estimation is the basic negative binomial model for segregation against the counts of homicides. As expected, the coefficient of income segregation has a large positive effect on homicides (IRR 3.1; CI 95% 2.5–3.9). The inclusion of other socioeconomic variables into the model attenuated the effect of segregation on homicide. However, the coefficient remained positive and significant (IRR 1.5; CI 95% 1.1–2.0).

**Table 4:** IRR from the estimation for socioeconomic segregation and homicides in 2010. Negative binomial model (exposure: population).

	Model 1	IRR (95% CI)	Model 2	IRR (95% CI)
SSI	3.1	(2.5–3.9)	1.5	(1.1–2.0)
				
Gini index			2.4	(2.0–3.0)
				
Unemployment			0.7	(0.5–1.0)
				
Complete secondary			0.8	(0.7–1.0)
				
Overcrowd_3b			1.1	(0.9–1.2)
				
Poverty rate			1.0	(0.7–1.4)
				
GDP per capita			1.2	(1.0–1.5)

*Age-adjusted homicides. *N* = 152.

This result reinforces Brazilian cities' unequal characteristics concerning the lack of public policies aimed at protecting individuals in conditions of social vulnerability. Such a situation leads to an increase in violence and can lead to serious consequences such as the high level of homicides observed throughout Brazil. This also means that beyond segregation, the entire social adverse context impact on the level of interpersonal violence in large Brazilian cities.

The observed correlation between socioeconomic segregation and homicides also strengths the findings of other similar works suggesting that segregation is harmful to health (Acevedo-Garcia, 2000; Ross and Dunn, 2001; Kramer and Hoguer, 2009; Nazari, Mahmoodi and Naieni, 2012; Marinacci et al., 2017; Chandola et al., 2018). In large Brazilian cities, income segregation has a central role in limiting social mobility across years and generations by determining access to education, health, public security, and employment opportunities. The poorest districts are also the ones that have the most deprived infrastructure and scarce supply of public goods and services. Consequently, people who live in more deprived neighborhoods are more likely to be exposed to violence. Just as the poor have constrained choices on where to live within a segregated city, poor people experience greater community violence burden (Wilkinson, 2004).

## 4 Conclusions

The search for understanding the relationship between socioeconomic segregation and homicides in cities can inform the design of public security policies aimed to improve public health. For this reason, we aimed to measure segregation in the 152 Brazilian cities with a population of over 100,000 inhabitants to investigate its relationship with homicide rates. It is an effort to provide relevant evidence to urban policymaking to promote social inclusion and improve population's health. The results suggest that a reduction in the current levels of segregation may play an important role in policies aimed to reduce violent crimes. Cities with a higher socioeconomic segregation level experienced higher homicide rates, with minimally adjusted estimates suggesting a nearly 50% increase in homicides per 1 SD greater segregation.

Additionally, variation in the socioeconomic segregation index itself showed it would be necessary to relocate 29.7% of the households in a typical large Brazilian city to reach a homogeneous spatial distribution concerning income across urban areas. In the 20 most segregated cities, at least 32.8% of low-income families would need to be relocated to achieve a homogeneous spatial distribution. We consider that this finding could have important implication for public policies in the large Brazilian cities.

Urban land use is limited by availability, affected by public housing policies, and subject to housing market pressures that may lead to gentrification and displacement (Comelli, Anguelovski and Chu, 2018). Homicides rates may increase as low-income populations experience deprivation and low living standards in these contexts of high segregation (Cufré, 2019). The analysis of the spatial distribution of income and homicide in the cities might importantly contribute to the understandig of the complex social factors associated with homicide. In this sense, we proposed to investigate the role of inequality and spatial displacement of the low-income population within urban agglomerations using the income dissimilarity index. The selected measure of income segregation was used in econometric models searching for explanation of city-level homicide rates aiming to produce new insights and to suggest potential policies for promoting social inclusion and desegregation.

This study has essential strengths related to the data source and the analytic approach. However, the used cross-section approach makes us cautious about making causal claims from reported findings of a possible effect of socioeconomic segregation on homicides. However, our results add to the well established causal relationship between inequalities and homicide. Segregation (measured by SSI) showed to be correlated with inequalities (measured by the Gini Index) in the studied Brazilian cities. But, while inequalities express the income-related social distances between a population, the segregation expresses the physical distance and the level of connection between social groups in the constrained urban space.

### Public policy implications

In a large and diverse country, such Brazil, the lack of planning and governance in large cities and urban areas has led the country to rates of lethal violence that are historically among the highest in the world (Briceno-León, Villaveces, and Concha-Eastman, 2008; Reichenheim et al., 2011; Cerqueira et al., 2019, p. 116).

In Brazil, social class in part determines the neighborhood of residence and the eventual opportunities for leisure and employment. The exclusion of peripheral populations is based on labor relations, poverty, and the generation of inequalities (Souza, 2001, 2005). Segregated urban areas create vulnerability for young people to prejudice and fear, and may increase engagement in illegal activities those resulting in violence. Such spatial patterns contribute to the increase of violent interpersonal and group conflicts in Brazil, mainly in favelas characterized, generally, as areas of risk often with disputed control by the militia, gangs, and criminal factions (Comelli, Anguelovski and Chu, 2018).

Segregation contributes to the degradation of spaces and the lack of physical, economic, and social infrastructure. These elements intensify the discrimination suffered by people living in needy areas, with the potential to generating interpersonal violence. For Cufré (2019), segregation itself is a form of symbolic violence, the effect of discrimination and inequality. Furthermore, the urban space is the result of social practices that are crucial for achieving social welfare. The lack of infrastructure in deprived neighborhoods can fuel violence. That is why it is so important that public authorities have medium and long-term plans to reduce segregation and inequalities among neighborhoods.

The social fragmentation caused by segregation and, consequently, the formation of new urban spaces around large cities creates “symbolic limits” for a given population, in which, on the one hand, the symbolic enclosure can be protective and on the other hand, given the power imbalance the poor can feel trapped. However, according to Alonso and Hita (2013), space alone does not generate violence. Specific social conditions, such as political, cultural, and economic issues are essential to foster marginalization, aggression, brutality, and even violent deaths.

In addition to these issues, problems related to violence and homicides need to be seen in the context of public health. Factors that promote violent deaths directly impact the population's health, health services demand and costs, institutional issues, social losses, physical and psychological trauma to individuals, etc. Violence is multifactorial and will only be resolved through the development of coordinated actions by government sectors and society to prevent its harmful effects. Therefore, based on the results presented here, we reinforce that policies that foster inclusion and desegregation may be promising to reduce the high rates of homicides in Brazilian cities.

## Declaration of interests

We declare no competing interests.

## CRediT authorship contribution statement

**Maria Izabel dos Santos:** Conceptualization, Methodology, Software, Data curation, Formal analysis, Writing – original draft, preparation, Visualization, and, Validation. **Gervásio Ferreira dos Santos:** Conceptualization, Formal analysis, and, Supervision. **Anderson Freitas:** Software, Visualization, Writing – review & editing. **J. Firmino de Sousa Filho:** Software, Formal analysis, Visualization, Writing – review & editing. **Caio Castro:** Writing – review & editing. **Aureliano S. Souza Paiva:** Software, Writing – review & editing. **Amélia A. de Lima Friche:** Writing – review & editing. **Sharrelle Barber:** Writing – review & editing. **Waleska Teixeira Caiaffa:** Writing – review & editing. **Maurício L. Barreto:** Conceptualization, Writing – review & editing, and, Supervision.

## Figures and Tables

**Table:** 

Region	Cities	Homicides (sum)	Population (sum)	Poverty rate (mean)	Per cap GDP (mean)	SSI (mean)	Gini (mean)	Unemp. Rate (mean)	Complete secondary (mean)
Midwest	8	2.795	7.851.353	21.1	18677.7	.274	.593	15.8%	40.3%
North	13	3.606	6.821.839	38.0	15753.3	.281	.578	10.9%	36.9%
Northeast	30	11.866	2.13e+07	45.0	8669.8	.326	.590	12.9%	39.5%
South	32	4.522	1.48e+07	15.3	17621.4	.243	.532	6.2%	37.0%
Southeast	69	14.787	5.89e+07	19.6	19610.8	.253	.540	8.1%	41.9%
Average	–	–	–	**25.4**	**16706.4**	**.269**	**0.554**	**9.3%**	**39.9%**
Total	**152**	**37.576**	**1.11e+08**			–	–	–	–

Source: Research Data Set.

## APPENDIX A

Regional Indicators of 152 cities by 5 Brazilian Macroregions.

## APPENDIX B

Correlation among the outcome and selected explanatory variables.

## Footnotes

1 DAG is an important tool for observational analysis of events. However, there are limitations regarding the construction of causal models, such as, for instance, the identification of immeasurable confounding variables. Despite this, this tool can generate useful conclusions even in situations where it is more difficult to identify a set of variables for controlling confounding.

2 Or in percentage terms as we present in the results, 0–100%.

3 In 2010, the official monthly minimum wage in Brazil was equal to R$ 510.00, thus income was dichotomized to define low income as below R$ 1020.00 monthly.

4 Except for the population of cities, included only as offset, an exposure variable.

5 Regarding Fig. 2d, household income up to ½ minimum wage is usually used to define families experiencing poverty as defined by the Brazilian government (Falcão and Costa, 2014).
